# A Cerebellar Framework for Predictive Coding and Homeostatic Regulation in Depressive Disorder

**DOI:** 10.1007/s12311-015-0708-2

**Published:** 2015-08-07

**Authors:** Dennis J. L. G. Schutter

**Affiliations:** Donders Institute for Brain, Cognition and Behaviour, Radboud University Nijmegen, Montessorilaan 3, 6525 HR Nijmegen, The Netherlands

**Keywords:** Cerebellum, Depressive disorder, Homeostasis, Monitoring, Predictive coding, Punishment, Reward

## Abstract

Depressive disorder is associated with abnormalities in the processing of reward and punishment signals and disturbances in homeostatic regulation. These abnormalities are proposed to impair error minimization routines for reducing uncertainty. Several lines of research point towards a role of the cerebellum in reward- and punishment-related predictive coding and homeostatic regulatory function in depressive disorder. Available functional and anatomical evidence suggests that in addition to the cortico-limbic networks, the cerebellum is part of the dysfunctional brain circuit in depressive disorder as well. It is proposed that impaired cerebellar function contributes to abnormalities in predictive coding and homeostatic dysregulation in depressive disorder. Further research on the role of the cerebellum in depressive disorder may further extend our knowledge on the functional and neural mechanisms of depressive disorder and development of novel antidepressant treatments strategies targeting the cerebellum.

## Introduction

Psychological wellbeing to a significant extend depends on the individual’s ability to deal with uncertainty [1]. Experience of uncertainty is inversely related to experiential feelings of control and results from actions that do not lead to the anticipated outcome. The mismatch between anticipated and actual outcome gives rise to a prediction error signal generated by the brain which is indicative for a disruption of internal bodily homeostasis [2]. Prediction error signals typically lead to a cascade of physiological and psychological processes that serve to re-establish equilibrium [3]. The idea of reducing uncertainty by optimizing predictability is known as the error minimization routine of predictive coding [2]. The error minimization routine is considered to form the basis of the organism’s ability to construct and update internal models that allow for successful adaptation under changing conditions [2]. Feedback-related reward and punishment signals arguably play an important role in the formation of internal prediction models and in shaping context appropriate behavior [4]. The fact that depressive disorder is characterized by hypersensitivity to punishment and hyposensitivity to reward suggests that in patients, reward and punishment signals provide suboptimal input for the error minimization routine to work properly. Support for this idea comes from findings showing that patients suffering from depression show abnormal neural responses to unexpected outcomes as well as context updating in response to feedback [5]. Atypical physiological response patterns to stress and abnormal biorhythms in patients with mood disorders further hint at a dysregulation of the neural circuitry concerned with bodily homeostasis. The subjective experience of negative mood and lack of control may thus represent a phenomenological proxy for problems in homeostatic regulatory function [6].

## Cerebellum and Predictive Coding

**Fig. 1:**
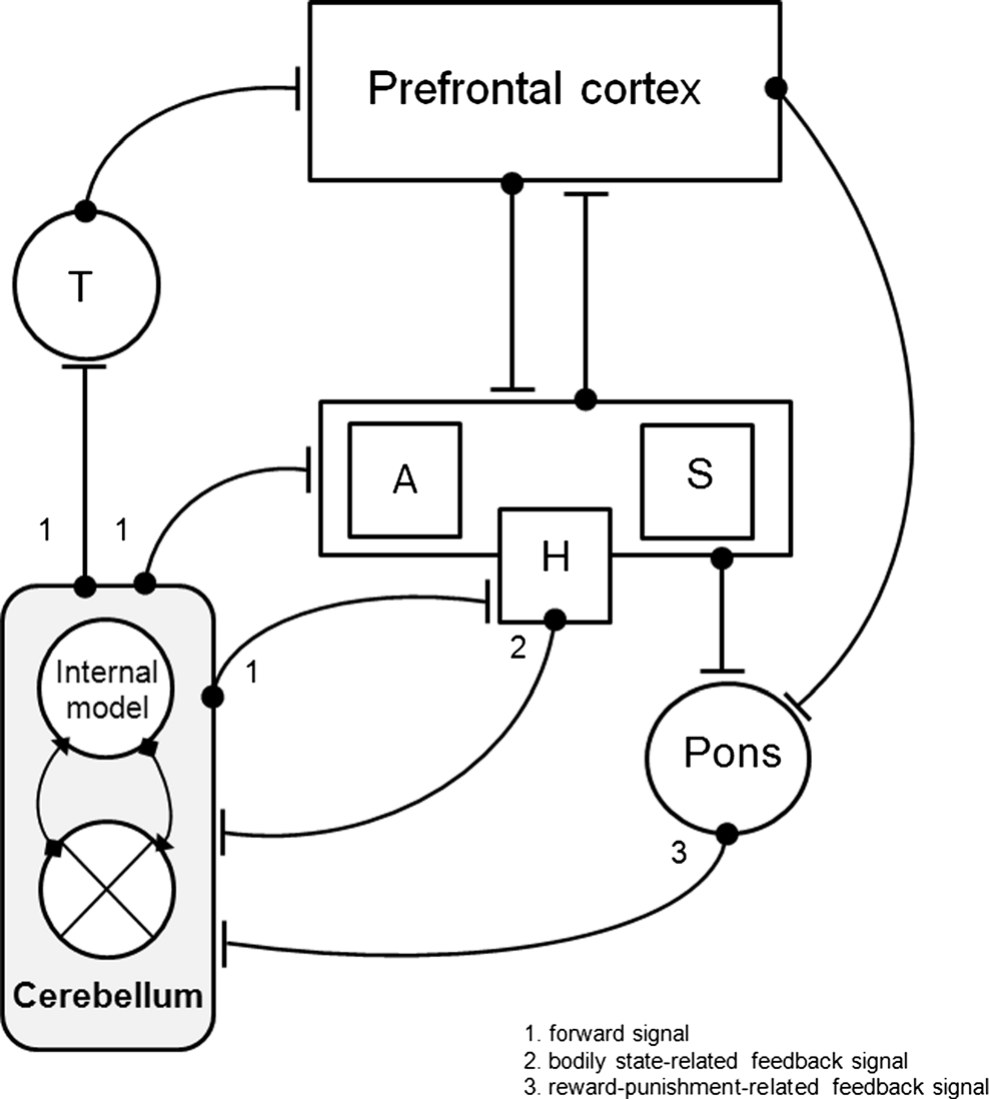
A functional neuroanatomical basis for the cerebellum in reward and punishment-related predictive coding and homeostatic regulation. *A* amygdala, *H* hypothalamus, *S* striatum, *T* thalamus

Central to predictive coding is the idea of the brain operating as a system which constructs experience-based inferential internal models of the world [2, 7]. While the role of the cerebellum in the context of adaptive control and predictive coding is established for the sensorimotor domain [8], current evidence indicates that this function extends to the non-motor domain as well [9]. Endocranial analyses of fossil humans demonstrate that in the course of evolution, expansion of the cerebral volume was paralleled by a quantitatively similar increase of cerebellar volume [10]. These volumetric increases have been attributed to growing social and cultural complexity [10]. While the cerebellum in modern man accounts for approximately 10 % of the total brain volume, this structure contains more than half of all neurons present in the human brain. These anatomical facts suggest that the cerebellum is a strong computational structure and plays a more prominent role in human behavior than previously thought [11]. This assumption is further strengthened by the reciprocal anatomical projections of the cerebellum to cortical and limbic areas. The posterior cerebellar hemispheres are connected to the cerebral cortex via the deep cerebellar nuclei and thalamus. Primate anatomical and human functional neuroimaging studies have shown that Crus I and II of the posterior cerebellar hemisphere are connected via the dentate nuclei to the association areas of the parietal and frontal cortex [12]. The anterior part of the cerebellum is linked to the subcortical punishment and reward structures of the brain, including the amygdala and striatum [9]. Furthermore, assuming a role for the cerebellum in the brain’s homeostatic functioning, the monosynaptic reciprocal connections to the hypothalamus are particularly notable [13]. The hypothalamus has a central role in governing metabolic, autonomic, and endocrine processes to maintain internal bodily homeostasis [6]. In addition, afferent connections from the cortical and limbic areas via the pontine nuclei located in the brainstem provide a closed circuit of cerebello-cortical and cerebello-limbic loops [14]. In fact, damage to the cerebellum can lead to dysfunctions in executive functioning, emotional instability, and mood [9]. Abnormal cerebellar activity associated with the processing of emotional relevant information in patients with depressive disorder has been confirmed in a meta-analysis of functional neuroimaging studies [15]. In addition, reduced cerebellar volumes in patients suffering from depressive disorder lend further support for cerebellar involvement in negative mood states [16]. These findings are in line with results showing an inverse parametric association between cerebellar volumes and neurotic personality traits in a non-clinical sample of volunteers [17]. The link between cerebellar volume and neuroticism hints at a possible role of the cerebellum in the vulnerability to experience negative affect and mood disorders. However, the associative nature of the study does not allow inferences on the directionality of the correlation. More direct evidence for cerebellar involvement in non-motor functions and mood comes from non-invasive brain stimulation studies in healthy volunteers [18]. Disruptive transcranial magnetic stimulation (TMS) applied to the cerebellum increases self-reports of negative mood as a result of impaired emotion regulation, while administering facilitatory TMS to the cerebellum increases positive mood and attentional biases for appetitive stimuli [18]. Recordings of distinct anterior scalp-recorded theta (4–7 Hz) oscillations following excitation of the human cerebellum with single-pulse TMS indicate the existence of a functional link between the cerebellum and the cerebral cortex [19]. Interestingly, theta oscillations are part of the cortico-limbic routines implicated in error monitoring and context updating. In further support, electric stimulation of the deep cerebellar nuclei elicits distinct theta oscillations located in limbic subdivisions of the prefrontal cortex in awake, behaving rats [20]. Taken together, these findings confirm the existence of reciprocal communication pathways between the cerebellum and cortico-limbic brain regions and provide a functional neuroanatomical basis for reward and punishment-related predictive coding and mood regulation (Fig. 1).

## Cerebellar Predictive Coding and Depressive Disorder

Major depressive disorder is associated with volume reductions in the frontal cortex, hippocampus, and striatum [21]. In addition, abnormal patterns of activity in the frontal cortex, striatum, globus pallidus, amygdala, and cerebellum have been observed [15]. These meta-analytic results support the view that depressive disorder involves abnormalities in the reward-punishment-related limbic circuits paralleled by dysfunctional brain structures implicated in top-down regulation. Further evidence for compromised regulation capacity and top-down regulation in depressive disorder was demonstrated in a recent meta-analysis which found lower functional connectivity between frontal and limbic structures [22]. The same study also showed that depressed patients had lower connectivity between parietal and frontoparietal regions involved in attending to the external environment but hyperconnectivity in the default network associated with internal self-referential thoughts and feelings [22]. Furthermore, the cerebellum was hypoconnected to the parietal cortex, a finding which concurs with the proposed cerebellar role in reality monitoring and predictive coding.

In agreement with conventional motor theories that conceptualize the cerebellum as a learning machine, three computational primitives for understanding the relations between homeostasis, predictive coding, and depressive disorder are important [10, 14]. Firstly, an internal forward model that is able to predict/anticipate the consequences of behavior. Secondly, an internal feedback model that is able to compare and detect mismatches between predicted and actual outcomes. Thirdly, an error minimization routine that actively modifies the internal forward and feedback models enabling more accurate predictions of the environment. From this viewpoint, behavioral adaptive responses involve the cerebellum performing Bayesian probability modeling that uses reward and punishment signals as inputs to update the priors to minimize uncertainty and regain bodily homeostasis [2]. Results from functional neuroimaging studies show that cerebellar activity correlates with error monitoring and probabilistic inferences in decision-making and context updating [23].

According to the uniform cerebellar transform function, the homogenous microstructure of the cerebellum allows for the processing of multimodal input signals originating from the rich cerebellar connections with cortical and limbic parts of the brain [9]. Anxiety and depression can be viewed as phenomenological manifestations of disrupted bodily homeostasis and uncertainty that prompts the cerebellum to update the priors of the internal model to minimize prediction errors. The conceptual framework predicts that in line with the universal cerebellar transform function, neuroticism and mood disorders are associated with problems in updating the internal model. Problems with updating the priors of the internal model will affect the prediction error minimization routine and contribute to feelings of uncertainty and loss of control. This view builds upon the central idea that the cerebellum is important for synchronizing cortical cognitive and limbic motivational information processing streams to fit contextual demands. In addition, the latter also gives a possible mechanistic account for why abnormalities in the cerebellar transform function could result in disorganized thought and feelings [9]. Abnormalities in cerebellar predictive coding may offer a theoretical framework to explain at least in part why anxiety and depression are associated with subjective reports of experiencing loss of control and feelings of helplessness. Finally, the present framework may provide a starting point for developing novel non-invasive brain stimulation protocols for the treatment of depressive disorder by targeting the cerebellum [18].

In conclusion, research has been discussed in support of the idea that the cerebellum contributes to reward- and punishment-related predictive coding and plays a role in the regulation of bodily homeostasis which is proposed to be dysfunctional in depressive disorder.
